# Ohio State Dental Society

**Published:** 1868-05

**Authors:** 


### Editorial.

### OHIO STATE DENTAL SOCIETY.

In pursuance of the passage of the law to *regulate* the practice of Dentistry in the State of Ohio, a called meeting of the State Society was held in the city of Columbus on the 20th inst., with the view of organizing to carry out the provisions of that law — at least so far as deciding upon the qualifications of a certain class of persons to practice Dentistry is concerned.

The main feature of this work was the appointment of a State Board of Examiners, consisting of five persons, from such points as will represent the different sections of the State as well as possible.

The Board is composed of the following persons: J. Taft, Cincinnati; W. P. Horton, Cleveland; F. H. Rehwinkle, Chillicothe; M. DeCamp, Mansfield; C. H. Harroun, Toledo.

The first meeting of the Board will be held early in July, of which timely notice will be given. Twenty-five dollars was fixed as the certificate fee, to be paid into the treasury of the society at the time of the reception of the certificate. All persons entering the practice of the profession, in this State after the passage of the law, must have a diploma from a Dental College, or a certificate of qualification from the Board of Examiners, or be subject to the penalties of the law.

There are very many excellent operators in the State who are not subject to the requirements of this law, at least till January 1st, 1873, who have not a diploma from a Dental College, whom it would be exceedingly gratifying to see go forward and avail themselves of the evidence of qualifications contemplated in this law. It would, in many communities, give them a position they could not otherwise attain; and would certainly in a marked degree, have that effect, so far as their standing in the profession, is concerned. Every truly professional man will honor the palpable evidence of thorough attainments in his professional brother. He who is wholly careless in reference to his position and standing in his profession is a drone.

The first meeting of the Board will be held at Columbus. All persons intending to appear for examination should notify some member of the Board at as early a day as possible.

The proceedings of the society of the 20th will be published in the June No. T.

